# High Expression of Long Non-Coding RNA TMCO1-AS1 is Associated With Poor Prognosis of Hepatocellular Carcinoma

**DOI:** 10.3389/fmolb.2022.814058

**Published:** 2022-01-24

**Authors:** Xuelian Huang, Sicong Zhu, Kelin Zhang, Wenliang Tan, Yajin Chen, Changzhen Shang

**Affiliations:** ^1^ Guangdong Provincial Key Laboratory of Malignant Tumor Epigenetics and Gene Regulation, Sun Yat-sen Memorial Hospital, Sun Yat-sen University, Guangzhou, China; ^2^ Department of Anesthesiology, Sun Yat-sen Memorial Hospital, Sun Yat-sen University, Guangzhou, China; ^3^ Department of Surgical Intensive Care Unit, Sun Yat-sen Memorial Hospital, Sun Yat-sen University, Guangzhou, China; ^4^ Department of Hepatobiliary Surgery, Sun Yat-sen Memorial Hospital, Sun Yat-sen University, Guangzhou, China

**Keywords:** long non-coding RNA, hepatocellular carcinoma, TMCO1-AS1, TCGA, prognosis

## Abstract

**Background:** The molecular pathways along with the clinical significance of long non-coding RNAs (lncRNAs) in hepatocellular carcinoma (HCC) remain uncertain. Our study sought to identify and characterize lncRNAs associated with HCC.

**Methods:** LncRNA TMCO1-AS1 was identified by differential expression analysis, receiver operating characteristic (ROC) analysis, and univariate analysis using RNA sequencing and clinical information of HCC from the public database. Then clinical correlations and survival analysis were conducted to further appraise the prognostic significance of lncRNA TMCO1-AS1 in HCC. Hepatoma and adjoining normal tissues from 66 patients who received surgical operation at our center were used to verify the results of the bioinformatics analysis. A survival prognostic model was established combining TMCO1-AS1 expression and other clinical characteristics.

**Results:** Bioinformatics analysis showed the aberrant high expression of TMCO1-AS1 in HCC tissue. TMCO1-AS1 expression was positively correlated with alpha-fetoprotein (AFP) level, vascular invasion, tumor stage, as well as tumor differentiation. Moreover, survival analysis found a significant inverse association between the expression of TMCO1-AS1 and the survival of patients with HCC. Cox analysis indicated that TMCO1-AS1 was an independent factor for HCC prognosis. Analysis of the HCC tissues from patients at our center provided results similar to those of the bioinformatics analysis. Risk models for overall survival (OS) and recurrence-free survival (RFS) incorporating TMCO1-AS1 exhibited better sensitivity and specificity than using clinical characteristics alone.

**Conclusion:** High TMCO1-AS1 expression is significantly correlated with the unfavorable poor prognosis of HCC, indicating its potential of being a novel prognostic marker for HCC.

## Introduction

Hepatocellular carcinoma (HCC) is generally acknowledged to be amongst the most prevalent digestive malignant tumors on a global scale. Recently, remarkable advancements have been achieved in the treatment of HCC patients, including local and systemic treatments, which have prolonged the survival of patients with HCC (Dhir et al., 2016; Reig et al., 2018). However, HCC is characterized by high invasiveness, metastasis, and frequent recurrence making the prognosis of patients with HCC far from satisfactory (Siegel et al., 2018). As such, identification of novel indicators or biomarkers of HCC may assist in the treatment of patients and help understand the underlying mechanisms of HCC.

Long non-coding RNA (lncRNA) are comprised of RNA transcripts whose length exceed 200 nucleotides but lack identifiable open reading frames. Growing studies have confirmed that lncRNAs participate in the proliferation, differentiation, apoptosis, and metastasis of many human cancers (Gibb et al., 2011; Bhan et al., 2017). In addition, the current relative study reveals that lncRNAs could act as tumor suppressor genes or oncogenes, thus exerting extensive and complicated roles in the regulation of the occurrence and progression of tumors (Maruyama and Suzuki, 2012). With advances in microarray technology and high-throughput RNA sequencing, numbers of dysregulated and expressed lncRNAs that participate in various processes of HCC have been identified (Da Sacco et al., 2012; Klingenberg et al., 2017). For example, lncRNA HOTTIP is shown to be implicated in the tumorigenesis and metastasis of HCC, and its overexpression predicts a poor outcome (Quagliata et al., 2014; Tsang et al., 2015). The lncRNA MATAL1, which is abnormally upregulated in HCC tissues, promotes the progression of HCC by suppressing ZEB1 expression *via* regulating miR-143-3p (Chen et al., 2017). MEG3 was the first lncRNA identified to function as a tumor suppressor gene; it interacts with P53 to inhibit tumor proliferation and its expression in HCC is low (Braconi et al., 2011). However, although many lncRNAs have been identified the biological functions of most remain unknown.

In this study, a novel lncRNA TMCO1-AS1 with aberrant expression in HCC was identified through bioinformatics analysis. The value of TMCO1-AS1 in HCC diagnosis and prognosis was evaluated by analyzing the gene expression matrix of The Cancer Genome Atlas (TCGA), and TMCO1-AS1 expression was demonstrated to be negatively correlated with survival of HCC patients. Furthermore, the data of HCC patients treated at our center was used to confirm the results of the bioinformatics analysis, and a prognostic model was built to improve the accuracy of prognosis prediction of HCC patients. Overall, our data indicate that lncRNA TMCO1-AS1 could serve as a promising novel prognostic marker for HCC.

## Materials and Methods

### Data Sources

The gene expression matrix (TPM format) of liver cancer was acquired by data download from TCGA (https://cancergenome.nih.gov/) using the R programming language (r-project.org). RNA sequencing (RNA-seq) data of 377 hepatoma tissues as well as 50 adjoining normal tissues from 377 patients were obtained and normalized into log_2_(TPM+1). In addition, the clinical data of the 377 patients were acquired as well. Patients with incomplete clinical and survival data were excluded. At last, a total of 314 patients with 364 tissue samples (314 hepatoma tissues and 50 adjoining normal tissues) were incorporated in the present research.

### Differential Expression Analysis

RNA-seq data were compared between 50 pairs of hepatoma tissues and corresponding adjacent tissues to identify the differentially expressed lncRNAs (DElncRNAs) using the “limma” package of R. False discovery rated (FDR) method was utilized for the purpose of adjusting the *p* values for several tests. The fold-change (FC) values of each lncRNA were calculated. A lncRNA was considered to have a significant differential expression when log_2_|FC| > 1.0 and *p* < .05.

### Evaluation of the Prognostic and Diagnostic Significance of Differentially Expressed lncRNAs for Hepatocellular Carcinoma

The expression profiles of the DElncRNAs were acquired, and in combination with survival prognostic information, their prognostic values were evaluated. Univariate regression was used to investigate if the expression levels of DElncRNAs were closely related to overall survival (OS) and recurrence-free survival (RFS). Receiver operating characteristic (ROC) analysis was conducted to evaluate the sensitivity and specificity of the DElncRNAs in diagnosing HCC. The following were the parameters that determine the area under the ROC curve (AUC): .5–.7, poor evidence for diagnosis; >.7–.9, moderate evidence for diagnosis; >.9–1.0, high-quality diagnostic evidence; >.7–.9, moderate-quality diagnostic evidence; .5–.7, poor -quality diagnostic evidence.

### Establishment of a Prognostic Model for Hepatocellular Carcinoma Survival

A risk model for anticipating the outcome of patients with HCC was constructed by combining DElncRNA expressions and patient clinicopathological characteristics. The necessary minimum number of clinicopathological characteristics were used to establish the model through least absolute shrinkage and selection operator) (LASSO) regression analysis, and the regression coefficients of each prognostic factor were calculated. Clinical characteristics scores were defined as 1 or 0 in accordance with different clinicopathological features. Utilizing equation as follows, we successfully calculated the risk score: Risk score = Exp × C_DElncRNA_ + S_1_ × C_1_+S_2_ × C_2_ + … + S_N_ × C_N_ (“Exp” means the expression quantity of the DElncRNA, “C” denotes the regression coefficient calculated by Lasso analysis, and “S” refers to the clinical characteristics scores). The median risk score was defined as a cutoff, and the patients were stratified into 2 groups with different risk levels. The comparison of OS and RFS was made between the grouped patients to evaluate the predictive ability of the models.

### Tissue Sample Collection

Hepatoma and corresponding adjacent normal tissues were acquired from 66 HCC patients who received surgical interventions at Sun Yat-sen Memorial Hospital, Sun Yat-sen University (Guangzhou, China) from 2015 to 2018. All patients had given informed consent for the collection of tissue samples for research purposes. Hepatocellular carcinoma was diagnosed for each patient by pathological examination of the operation samples. Preoperatively, none of the patients underwent any type of treatment before surgery (e.g., received radiotherapy, chemotherapy, immunotherapy). Normal tissues that were adjoining to the malignant tumor were obtained at a distance of 2.0 cm. Liquid nitrogen was used to preserve all of the samples from the surgery. At least 2 years of follow-up was required for each patient.

### Isolation of RNA and Quantitative Reverse Transcription-Polymerase Chain Reaction

Trizol (Takera, Japan) was utilized for the purpose of performing the isolation of the total RNA. Reverse transcription was conducted using A PrimeScript RT reagent Kit (Takara, Japan) was utilized for reverse-transcribing the isolated RNA, whereas SYBR Premix Ex Taq (Takara, Japan) was employed in cDNA amplification. PCR was performed on a CFX96 system (BIO-RAD, USA) at the temperature for 30 s at 95°C, followed by 40 cycles at 95°C for 5 s and 60°C for 20 s. Internal controls were implemented using GAPDH. The relative expression of TMCO1-AS1 was determined utilizing the 2^−ΔΔCT^ method. The primers had the following sequences: TMCO1-AS1 forward: 5′- GTT​TAG​CTT​GGG​TTT​GCC​GT -3′, reverse: 5′- AGC​GGC​CCA​CAA​CTA​ACT​C -3′; GAPDH forward: 5′- CCA​GAA​CAT​CAT​CCC​TGC​CT -3′, reverse: 5′- CCT​GCT​TCA​CCA​CCT​TCT​TG -3′.

### Statistical Analysis

Continuous variables were compared by one-way analysis of variance (ANOVA) or two-sided Student’s t-test, as deemed necessary, whereas comparisons of categorical variables were performed by Chi‐square test. The log-rank test, as well as the landmark analysis, were employed to contrast Kaplan–Meier curves. Univariable and multivariable analyses were conducted based on the Cox regression method. We conducted the receiver operating characteristic (ROC) analysis to examine the prediction capacity of the lncRNAs. All statistical analyses were implemented using SPSS (IBM Corporation). The volcano plot and heatmap were charted and analyzed utilizing ImageGP (http://www.ehbio.com/ImageGP/index.php). Differential expression analysis was performed using the “limma” package of R. LASSO analysis was conducted using the “glmnet” R package, and time-dependent ROC curves were drawn with the “survivalROC” R package. Statistical significance was judged to have been attained when *p* < .05.

## Results

### The Cancer Genome Atlas Database Analysis of lncRNAs Differentially Expressed Between Hepatocellular Carcinoma and Adjacent Tissue

**FIGURE 1:**
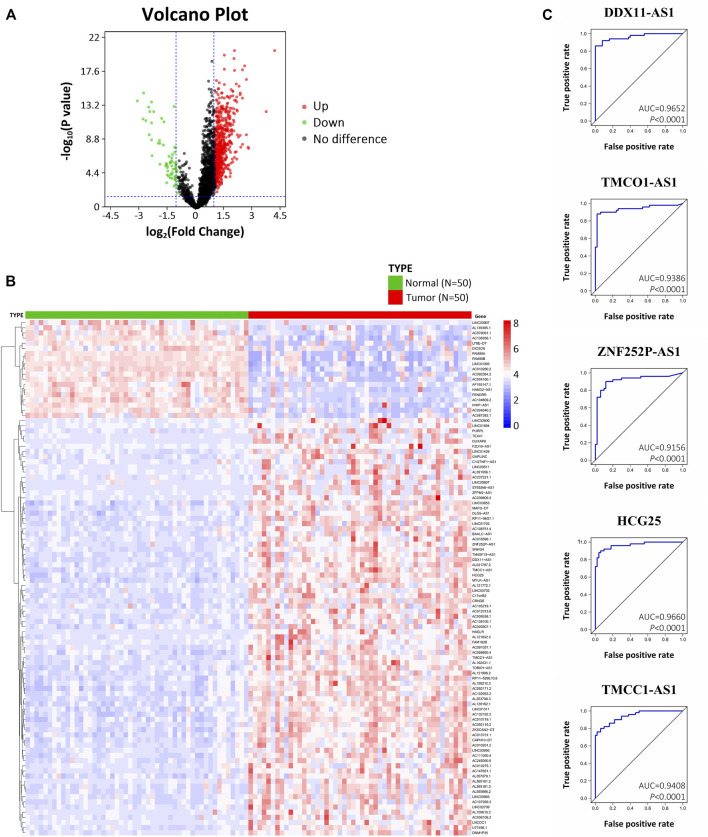
Differentially expressed lncRNAs in HCC based on bioinformatics analysis of TCGA. **(A)** Volcano plot illustrating the DElncRNAs between HCC tissues and adjacent normal tissues. **(B)** Heatmap showing the hierarchical clustering of the top 100 DElncRNAs. **(C)** ROC curves of five DElncRNAs (DDX11-AS1, TMCO1-AS1, ZNF252P-AS1, HCG25, TMCC1-AS1) with an AUC > .9.

With the criteria of log_2_|FC| > 1.0 and *p* < .05, 1,304 DElncRNAs were identified with 1,111 upregulated and 193 downregulated. Figure 1A showed the volcano plot of the DElncRNAs which was plotted based on differential expression analysis. The expression profiles of the top 100 DElncRNAs (81 up-regulated and 19 downregulate; Supplementary Table S1) were illustrated using a heatmap (Figure 1B). Based on DElncRNA patterns, tumor tissue could be distinguished from normal tissue.

### Diagnostic and Prognostic Value of Differentially Expressed lncRNAs

Univariate analysis was performed for OS and RFS to determine whether a DElncRNA was substantially correlated with HCC patients’ prognosis. With *p* < .05, 31 DElncRNAs were detected to be significantly correlated to OS, and 35 DElncRNAs were significantly correlated to RFS (Supplementary Table S2). Among them, there was a significant difference of OS and RFS associated with 15 DElncRNAs (DUXAP8, DDX11-AS1, TMCO1-AS1, ZNF252P-AS1, AC091057.1, TM4SF19-AS1, HCG25, RP11-98G7.1, SNHG4, AC012313.8, TMCC1-AS1, ZFPM2-AS1, AL357079.1, LINC02709, and AC099850.4), suggesting they have remarkable prognostic value for HCC patients.

ROC analysis was performed on the 15 DElncRNAs to detect whether the DElncRNA could serve as a biomarker to distinguish HCC tissue from adjacent normal tissue. Of the 15 DElncRNAs, 5 (DDX11-AS1, TMCO1-AS1, ZNF252P-AS1, HCG25, and TMCC1-AS1) exhibited an AUC > .9 (Figure 1C). These results indicate that the 5 DElncRNAs, which are all up-regulated in HCC, have marked diagnostic potential for HCC.

For further study, we selected lncRNA TMCO1-AS1 which has not been reported to be associated with HCC before.

### Expression and Clinical Correlation of TMCO1-AS1 in Hepatocellular Carcinoma

**FIGURE 2:**
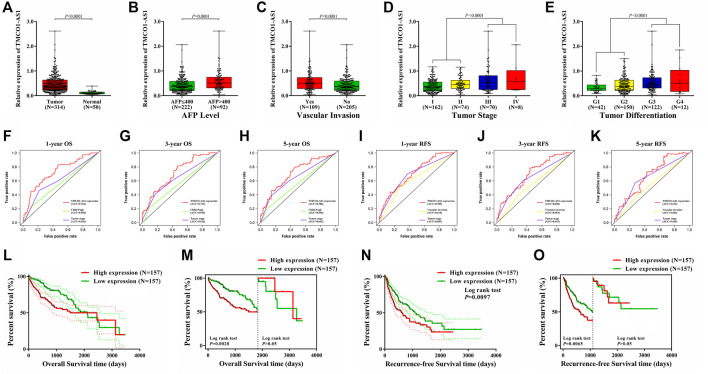
Dysregulation of TMCO1-AS1 in HCC and its prognostic significance for HCC using data of patients from TCGA. **(A)** The expression levels of TMCO1-AS1 in HCC and adjacent normal tissues. **(B)** TMCO1-AS1 expression in patients with high (AFP >400 μg/L) and low (AFP ≤400 μg/L) serum AFP levels. **(C)** TMCO1-AS1 expression in patients with and without vascular invasion. **(D)** TMCO1-AS1 expression in patients with different tumor stages. **(E)** TMCO1-AS1 expression in patients with different degrees of tumor differentiation. **(F–H)** Time-dependent ROC curves of the relations of clinical characteristics and 1-/3-/5-year OS. **(I–K)** Time-dependent ROC curves of the relations of clinical characteristics and 1-/3-/5-year RFS. **(L)** Kaplan-Meier curves for OS of HCC patients from TCGA with high and low TMCO1-AS1 expression. **(M)** Landmark analysis discriminating between OS before and after 5 years of follow-up. **(N)** Kaplan-Meier curves for RFS of HCC patients from TCGA with high and low TMCO1-AS1 expression. **(O)** Landmark analysis discriminating between RFS before and after 3 years of follow-up.

The expression of TMCO1-AS1 between 314 hepatoma tissues and 50 adjoining normal tissues from TCGA was compared. Figure 2A illustrates that TMCO1-AS1 expression in HCC tissues was obviously elevated in contrast with that in adjacent tissues (*p* < .0001). Median expression of TMCO1-AS1 was defined as a threshold point to classify the 314 HCC patients into low- and high-expression groups, and patient clinical data of the two groups were analyzed. Correlation analysis demonstrated that TMCO1-AS1 expression was considerably related to alpha-fetoprotein (AFP) level (*p* = .0029), vascular invasion (*p* = .0014), TNM stage (*p =* .0182) and tumor differentiation (*p* = .0016) (Table 1). The expression of TMCO1-AS1 in patients whose levels of AFP were >400 μg/L was substantially higher in contrast with patients whose levels of AFP were ≤400 μg/L (Figure 2B). Patients with vascular invasion had greater expression of TMCO1-AS1 than in HCC tissues compared to those without vascular invasion (Figure 2C). High expression of TMCO1-AS1 in HCC tissues was positively related to an advanced stage and poor differentiation (Figures 2D,E). However, TMCO1-AS1 expression had no associations with other features, for example, age, sex, race, virus hepatitis, alcoholic hepatitis, Child-Pugh classification, and cirrhosis (all, *p* > .05).

**TABLE 1:** Relationship between the expression levels of TMCO1-AS1 and clinicopathological characteristics in 314 HCC patients from TCGA.

Clinicopathological characteristics		TMCO1-AS1 expression		*χ* ^2^	*p* value
		Low (*N* = 157)	High (*N* = 157)		
Age (years)	≤60	70	75	.320	.5714
	>60	87	82		
Gender	Male	111	95	3.613	.0573
	Female	46	62		
Race	Yellow	69	68	.099	.9519
	White	80	82		
	Black	8	7		
AFP (µg/L)	≤400	123	99	8.855	.0029^a^
	>400	34	58		
Hepatitis virus infection	Yes	70	83	2.154	.1422
	No	87	74		
Alcoholic hepatitis	Yes	56	46	1.452	.2282
	No	101	111		
Child-Pugh	A	138	132	.952	.6213
	B	16	21		
	C	3	4		
Cirrhosis	Yes	57	64	.659	.4170
	No	100	93		
Vascular invasion	Yes	41	68	10.244	.0014^a^
	No	116	89		
Tumor stage	I	95	67	10.045	.0182^a^
	II	30	44		
	III	29	41		
	IV	3	5		
Tumor differentiation	G1	31	11	15.334	.0016^a^
	G2	78	72		
	G3	43	67		
	G4	5	7		

a *p* < .05.

### Relations Between TMCO1-AS1 Expression and Hepatocellular Carcinoma Prognosis Using The Cancer Genome Atlas

Cox analysis was conducted to appraise the value of TMCO1-AS1 for HCC prognosis. Univariate regression analysis showed that hepatitis virus infection (*p* = .0351), Child-Pugh score (*p* = .0153), tumor stage (*p* < .0001) and the levels of TMCO1-AS1 expression (*p* = .0079) were considerably correlated with OS. Moreover, vascular invasion (*p* = .0013), tumor stage (*p* < .0001) and TMCO1-AS1 expression (*p* = .0103) were significantly associated with RFS (Supplementary Table S3). Multivariate regression analysis indicated that TMCO1-AS1 independently acted as a predictive marker of HCC prognosis (Supplementary Table S4).

Time-dependent ROC analysis was performed to compare the prediction values of the prognostic parameters determined through the Cox analysis. The time-dependent ROC curves of 1-year OS for TMCO1-AS1 expression, Child-Pugh score, and tumor stage are shown in Figure 2F. The AUC for TMCO1-AS1 expression was .724, and the AUC for tumor stage and Child-Pugh score were .606 and .558. The AUC of 3-year OS for TMCO1-AS1, tumor stage and Child-Pugh score were .713, .636, and .559, respectively (Figure 2G). The AUC of 5-year OS was .708, which was larger than that for Child-Pugh score and tumor stage (Figure 2H). Thus, of the three factors TMCO1-AS1 exhibited the best ability to predict OS of patients with HCC. Similarly, the time-dependent ROC curves of 1-year RFS for TMCO1-AS1 expression, tumor stage and vascular invasion are displayed in Figure 2I. The AUC for TMCO1-AS1 expression was .705, and that for tumor stage and vascular invasion were .687 and .619, respectively. The AUC of 3-/5-year RFS for TMCO1-AS1 was larger than that for tumor stage and vascular invasion (Figures 2J,K). Thus, of the three factors TMCO1-AS1 exhibited the best ability to predict RFS of HCC patients.

Kaplan-Meier curves were drawn to analyze OS and RFS between HCC patients exhibiting low and high TMCO1-AS1 expression. In patients exhibiting elevated TMCO1-AS1 expression, the median OS time was 1,490 days, whereas in patients having low TMCO1-AS1 expression, their median OS time was 2,116 days (Figure 2L). Figure 2M showed a landmark analysis of OS before and after 5 years of follow-up. Within 5 years, patients in the high TMCO1-AS1 expression group exhibited obviously shortened OS time as opposed to patients in the low TMCO1-AS1 expression group (*p* = .0020). While after 5 years, no statistical differences were discovered between the OS of the two groups (*p* > .05). Patients having elevated TMCO1-AS1 expression had significantly shorter median RFS time compared to those with low TMCO1-AS1 expression (Figure 2N). Landmark analysis revealed the discrimination between tumor recurring before and after 3 years of follow-up, and a significant difference of RFS within 3 years was detected between patients exhibited low and high expression of TMCO1-AS1 (Figure 2O). Survival analysis showed that TMCO1-AS1 had a certain value in the prediction of HCC prognosis, especially for the early-term survival.

**FIGURE 3:**
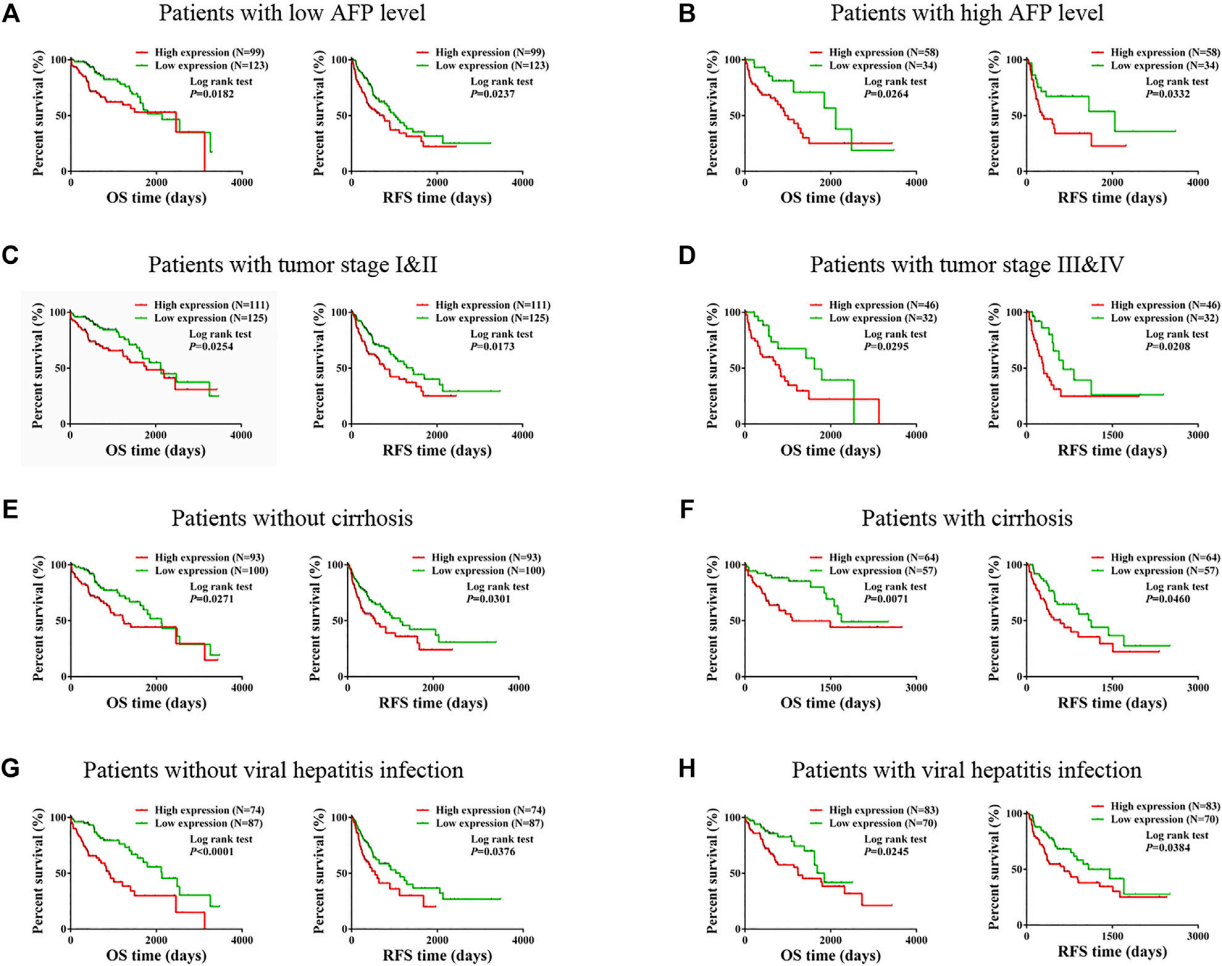
Prognostic value of TMCO1-AS1 expression in HCC patients with different characteristics. **(A)** Survival curves for OS and RFS of patients with AFP ≤400 μg/L. **(B)** Survival curves for OS and RFS of patients with AFP >400 μg/L. **(C)** Survival curves for OS and RFS of patients with tumor stage I-II. **(D)** Survival curves for OS and RFS of patients with tumor stage III-IV. **(E)** Survival curves for OS and RFS of patients without cirrhosis. **(F)** Survival curves for OS and RFS of patients with cirrhosis. **(G)** Survival curves for OS and RFS of patients without viral hepatitis infection. **(H)** Survival curves for OS and RFS of patients with viral hepatitis infection.

We especially carried out a subgroup analysis to thoroughly examine the prognostic significance of TMCO1-AS1 for HCC patients with different clinical characteristics. In accordance with the clinical characteristics including serum AFP level, tumor stage, liver cirrhosis and hepatitis virus infection, the patients with HCC obtained from TCGA were classified into various groups. Kaplan-Meier analysis indicated there was a statistical difference in OS and RFS of the patients classified under the group having the level of AFP as ≤400 μg/L (Figure 3A) in contrast with those having AFP > 400 μg/L (Figure 3B). In addition, statistical differences in OS and RFS were found between patients with and without cirrhosis (Figures 3C,D), patients with early-stage or advanced-stage disease (Figures 3E,F), and those with and without viral hepatitis (Figures 3G,H).

### Correlation of Clinical Variables and Survival Analyses of Patients at Our Center

**FIGURE 4:**
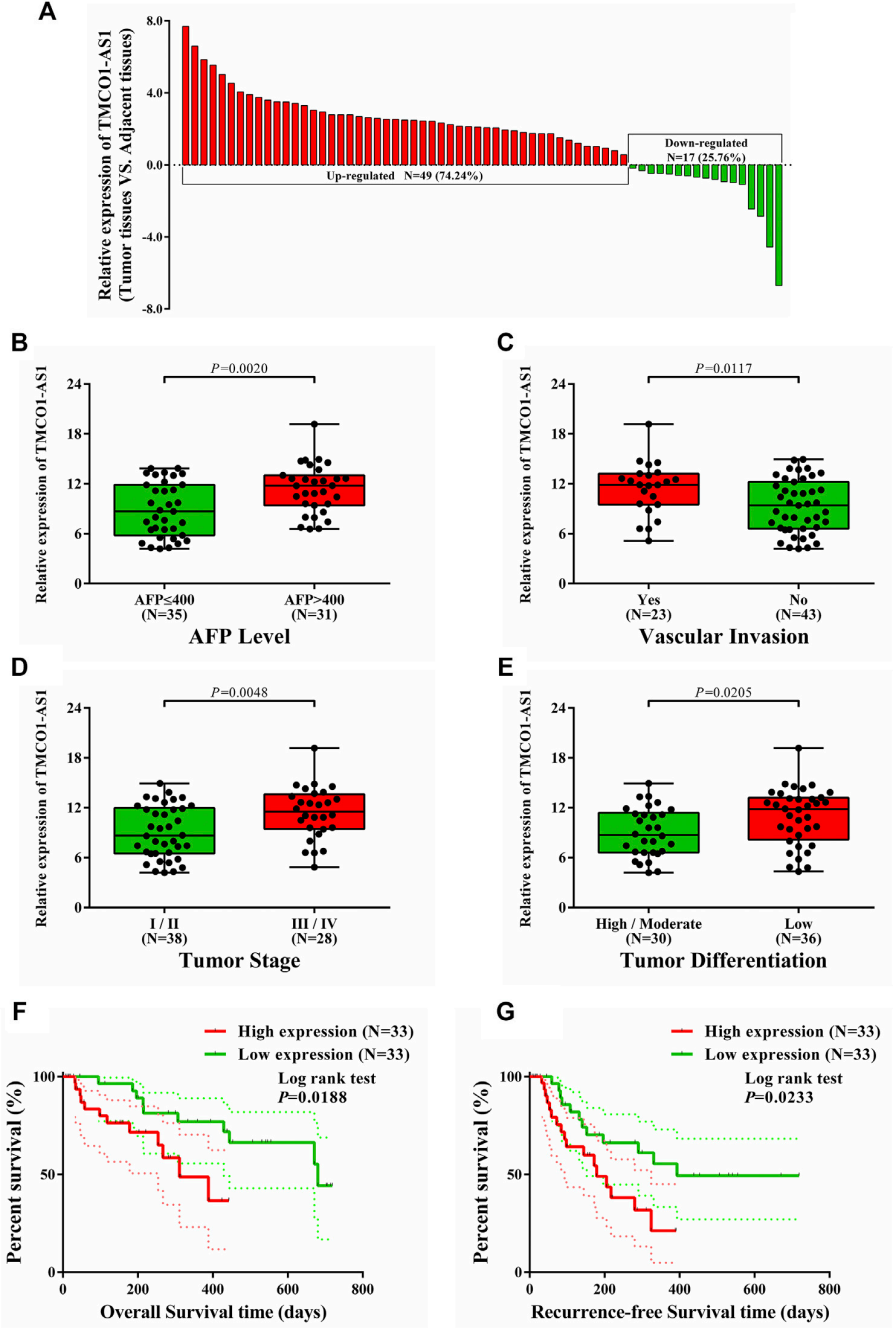
Relations between TMCO1-AS1 expression and clinical characteristics of HCC patients treated at our center. **(A)** Relative expression of TMCO1-AS1 in 66 HCC and adjacent normal tissues. **(B)** TMCO1-AS1 expression in patients with high and low serum AFP levels. **(C)** TMCO1-AS1 expression in patients with and without vascular invasion. **(D)** TMCO1-AS1 expression in patients with different tumor stages. **(E)** TMCO1-AS1 expression in patients with different degrees of tumor differentiation. **(F)** Kaplan-Meier curves for OS of patients with high and low TMCO1-AS1 expression. **(G)** Kaplan-Meier curves for RFS of patients with high and low TMCO1-AS1 expression.

The expression of TMCO1-AS1 was detected in 66 paired samples of hepatoma and adjoining normal tissues from patients at our center that received surgery. Figure 4A depicted that the levels of TMCO1-AS1 expression were significantly increased in 74% of HCC tissues (49/66) in contrast with the matching adjoining normal tissues.

Correlation analysis using data from the patients treated at our center showed that serum AFP level (*p* < .0001), vascular invasion (*p* = .0367), tumor stage (*p* = .0267), and differentiation (*p* = .0138) were significantly associated with TMCO1-AS1 expression (Table 2). Furthermore, patients having an elevated AFP level and vascular invasion had a high expression level of TMCO1-AS1 in tumor tissue (Figures 4B,C). And TMCO1-AS1 was overexpressed in tumor tissue of patients with advanced tumor stage and attenuated tumor differentiation (Figures 4D,E). All patients from our center were followed up for more than 2 years. Kaplan-Meier analysis illustrated that OS and RFS of patients exhibiting elevated TMCO1-AS1 expression were obviously shortened as opposed to that of patients exhibiting low TMCO1-AS1 expression, which were in line with the findings from bioinformatics in TCGA (Figures 4F,G).

**TABLE 2:** Relationship between the expression levels of TMCO1-AS1 and clinicopathological characteristics in 66 HCC patients from our center.

Clinicopathological characteristics		TMCO1-AS1 expression		*χ* ^2^	*p* value
		Low (*N* = 33)	High (*N* = 33)		
Age(years)	≤60	20	22	.262	.6088
	>60	13	11		
Gender	Male	28	29	.129	.7198
	Female	5	4		
AFP (μg/L)	≤400	22	13	4.927	.0264^a^
	>400	11	20		
Hepatitis virus infection	Yes	27	26	.096	.7569
	No	6	7		
Alcoholic hepatitis	Yes	13	10	.601	.4383
	No	20	23		
Child-Pugh	A	32	31	.349	.5546
	B/C	1	2		
Cirrhosis	Yes	17	16	.061	.8055
	No	16	17		
Tumor size (cm)	≤5	9	12	.629	.4279
	>5	24	21		
Vascular invasion	Yes	7	16	5.405	.0201^a^
	No	26	17		
Tumor stage	I/II	24	14	6.203	.0128^a^
	III/IV	9	19		
Tumor differentiation	High/Moderate	19	11	3.911	.0480^a^
	Low	14	22		

a *p* < .05.

### Construction of Risk Score System for Hepatocellular Carcinoma

**FIGURE 5:**
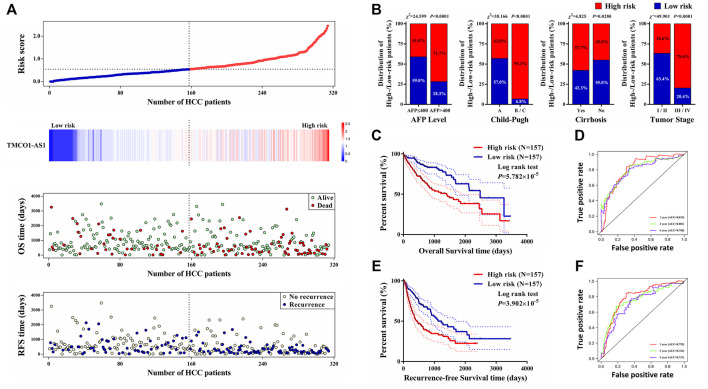
A prognostic model for OS and RFS of HCC patients from TCGA. **(A)** Risk score distribution, TMCO1-AS1 expression, and OS and RFS of patients in the high- and low-risk group. **(B)** Comparison of the distributions of patients with different characteristics in the high- and low-risk groups. **(C)** Kaplan-Meier OS curves for patients in the high- and low-risk groups. **(D)** Time-dependence ROC curves of the risk score for OS. **(E)** Kaplan-Meier RFS curves for patients in the high- and low-risk groups. **(F)** Time-dependence ROC curves of the risk score for RFS.

Data of 314 HCC patients from TCGA were used to build a model for predicting OS and RFS. The clinical characteristics score of 1 was assigned to the features of age >60 years, male, Child‐Pugh class B/C, liver cirrhosis, serum AFP >400 μg/L, vascular invasion, tumor stage III–IV, and tumor grade 3–4. The score of 0 was assigned to age ≤60 years, female, Child‐Pugh class A, no cirrhosis, serum AFP ≤400 μg/L, no vascular invasion, tumor stage I–II, and tumor grade 1–2. Using Lasso analysis, risk prognostic models were established for OS and RFS by combining the expression of TMCO1-AS1 and three clinical characteristics. The risk scores of all patients from TCGA were calculated using the following formula: Risk score = .9066 × Exp_TMCO1-AS1_ + .1207 × S_AFP_ + .2787 × S_Child_ + .0882 × S_Cirrhosis_ + .6105 × S_Stage_ (S is the score [0 or 1] of the subscripted variable and Exp is the expression of the lncRNA). Patients were classified into low- and high-risk groups according to their median value risk scores. As shown in Figure 5A, TMCO1-AS1 expression exhibited a positively association with the risk score. Moreover, the OS and RFS of the high-risk group patients were poorer in contrast with that of the low-risk group patients. Based on the risk model, a larger proportion of patients with high serum AFP, Child-Pugh class B/C, liver cirrhosis, and advanced tumor stage were defined as high-risk (Figure 5B). Kaplan-Meier analysis demonstrated that the median OS of high-risk patients (1,397 days) was substantially shortened in contrast with that of low-risk patients (2,486 days) (Figures 5C,D). The time-dependent AUC of the risk model’ OS for 1-/3-/5-year was .815, .801, and .788, respectively, which was larger than the 1-/3-/5-year AUC for TMCO1-AS1 expression, Child-Pugh class, and tumor stage. Comparable findings were observed when RFS was analyzed; the high-risk group patients exhibited considerably shortened RFS in contrast with the low-risk group patients (Figure 5E). The AUC 1-/3-/5-year RFS for the risk score was .792, .762, and .731, suggesting a good predictive value of the model (Figure 5F). For both OS and RFS, the risk model exhibited better sensitivity and specificity for the prediction than any single clinical feature. The clinical data and expression profiles of 66 HCC patients from our center was utilized to further verify the prediction ability of the risk model in HCC prognosis, and the results were similar to those obtained by analyzing data from TCGA (Supplementary Figure S1).

## Discussion

Hepatocellular carcinoma is the seventh most frequent malignant tumor and has been ranked as the third major contributor to cancer-associated fatalities globally (Bray et al., 2018). Although recent developments of locoregional and molecular targeted therapies have been reported to provide a survival benefit, surgery is still a curative option (Kudo, 2018; Raoul et al., 2018; Raoul et al., 2019). However, because of a lack of obvious symptoms a considerable proportion of patients received their diagnosis at an advanced stage resulting in a poor prognosis (Fan, 2012). Moreover, the postoperative recurrence rate of patients who undergo liver resection is as high as 70% (Llovet et al., 2005). Thus, early diagnosis and prognostic markers are important in the treatment of HCC, as is timely following-up after surgery.

Alpha-fetoprotein is a traditional cancer biomarker that is used to monitor HCC treatment and recurrence; however, 35–45% of HCC patients have low serum AFP levels (<20 μg/L) (Sherman et al., 1995; Cedrone et al., 2000; Trevisani et al., 2001). Conventional prognostic systems such as Barcelona Clinic Liver Cancer (BCLC) stage and TNM classification are valuable in the prediction of HCC patients’ survival status (Imai et al., 2014; Lee et al., 2014). However, HCC patients with the same BCLC stage or TNM classification may have different outcomes, illustrating the limitations of these systems for patients with HCC.

Tumorigenesis is commonly the result of the abnormal expression of a specific gene of genomes. Currently, lncRNAs are an active area of research because their expression is frequently dysregulated in cancers and their biological function may be oncogenic and tumor suppressor activity. Thus, the possibility that lncRNAs can serve as diagnostic and prognostic biomarkers has attracted increasing attention (Wang and Tran, 2013). A growing number of lncRNAs are demonstrated to play major parts in the process of HCC tumorigenesis. For instance, the plasma level of lncRNA HULC (highly upregulated in liver cancer) has been reported to be useful for HCC diagnosis and prognosis (Xie et al., 2013). Urothelial carcinoma associated-1 (UCA1) has an indispensable proliferation-related role in HCC *via* the Hippo signaling pathway, and it has been shown to be useful for predicting the prognosis (Qin et al., 2018). A lncRNA down-regulated in liver cancer stem cells (lncDILC) has been shown to interfere with NF-κB-mediated IL-6 expression *via* inhibiting STAT3 signaling, and thus has prognostic value for patients with HCC (Wang et al., 2016).

Bioinformatics analysis is an accurate and effective method to explore the functionality of genes in certain diseases, especially in malignancies. Increasing numbers of lncRNAs with an aberrant expression that are involved in HCC development and progression are being identified. In our study, DElncRNAs were distinguished and screened by differential expression analysis using the expression profiles of HCC tissues from TCGA. Subsequently, we identified five DElncRNAs with significant diagnostic and prognostic value in HCC. Among the five DElncRNAs, four have recently been shown to perform a function in the onset and progression of HCC. The lncRNA DDX11-AS1 suppresses the LATS2 protein *via* interacting with EZH2 and DNMT1 in HCC cells and may function as an oncogene, and the expression of DDX11-AS1 is correlated with a poor HCC prognosis (Li et al., 2019). The lncRNA ZNF252P-AS1 has been shown to be significantly associated with patients’ OS with hepatitis B virus (HBV)-related HCC (Zhao et al., 2020). The lncRNA HCG25 is aberrantly expressed in HCC tissue, and has a significant diagnostic value for HCC (Shi et al., 2018). Prior studies have reported that TMCC1-AS1 is related to autophagy, and its expression is negatively correlated with HCC prognosis (Cui et al., 2017; Zhao et al., 2018; Deng et al., 2020).

TMCO1-AS1, also referred to as RP11-466F5.8 or ENSG00000224358, is antisense RNA located on chromosome 1 with a length of 2,148 bp. In our study, we identified this novel lncRNA, which has not been previously reported associated with HCC, *via* bioinformatics analysis. TMCO1-AS1 was found to be significantly overexpressed in HCC tissues, ROC analysis showed an excellent prognostic value for HCC, and survival analysis revealed that TMCO1-AS1 expression was inversely correlated with OS and RFS of HCC patients. TMCO1-AS1 was shown to independently serve as a risk factor for HCC, and it had a better prognostic value than other clinical features such as Child-Pugh class, cirrhosis, vascular invasion, and tumor stage.

In this study, we also developed a novel risk model for predicting OS and RFS using TMCO1-AS1 expression and other clinical characteristics. OS and RFS differed considerably between the low- and high-risk group patients, and the prognostic value for OS and RFS was verified by time-dependent ROC analysis. Moreover, the prediction ability of the constructed risk model was shown to be better than that of other clinical features, and thus may serve as a novel method for establishing the prognosis for the patients with HCC. Notably, TMCO1-AS1 expression was positively related to the HCC risk score. Overall, our findings indicated that TMCO1-AS1 is involved in the occurrence and progression of HCC and influences patients’ prognosis. However, the exact biological function and molecular mechanism of TMCO1-AS1 require further study.

## Conclusion

In summary, we identified a novel lncRNA, TMCO1-AS1, in HCC. High expression of TMCO1-AS1 in HCC tissues was correlated with poorer OS and RFS. Thus, the lncRNA TMCO1-AS1 could serve as a valuable prognostic marker for HCC patients.

## Data Availability

The original contributions presented in the study are included in the article/Supplementary Material, further inquiries can be directed to the corresponding authors.

## Supplementary Material

Supplementary Figure 1 A prognostic model for OS and RFS of HCC patients from our center. **(A)** Risk score distribution, TMCO1-AS1 expression, and OS and RFS of patients in the high- and low-risk group. **(B)** Comparison of the distributions of patients with different characteristics in the high- and low-risk groups. **(C)** Kaplan-Meier OS curves for patients in the high- and low-risk groups. **(D)** Kaplan-Meier RFS curves for patients in the high- and low-risk groups.
