# Supplementary figures and images for: High Expression of Long Non-Coding RNA TMCO1-AS1 is Associated With Poor Prognosis of Hepatocellular Carcinoma
Source: Front Mol Biosci. 2022 Jan 24;9:814058. doi: 10.3389/fmolb.2022.814058 (PMC8819098; doi:10.3389/fmolb.2022.814058)

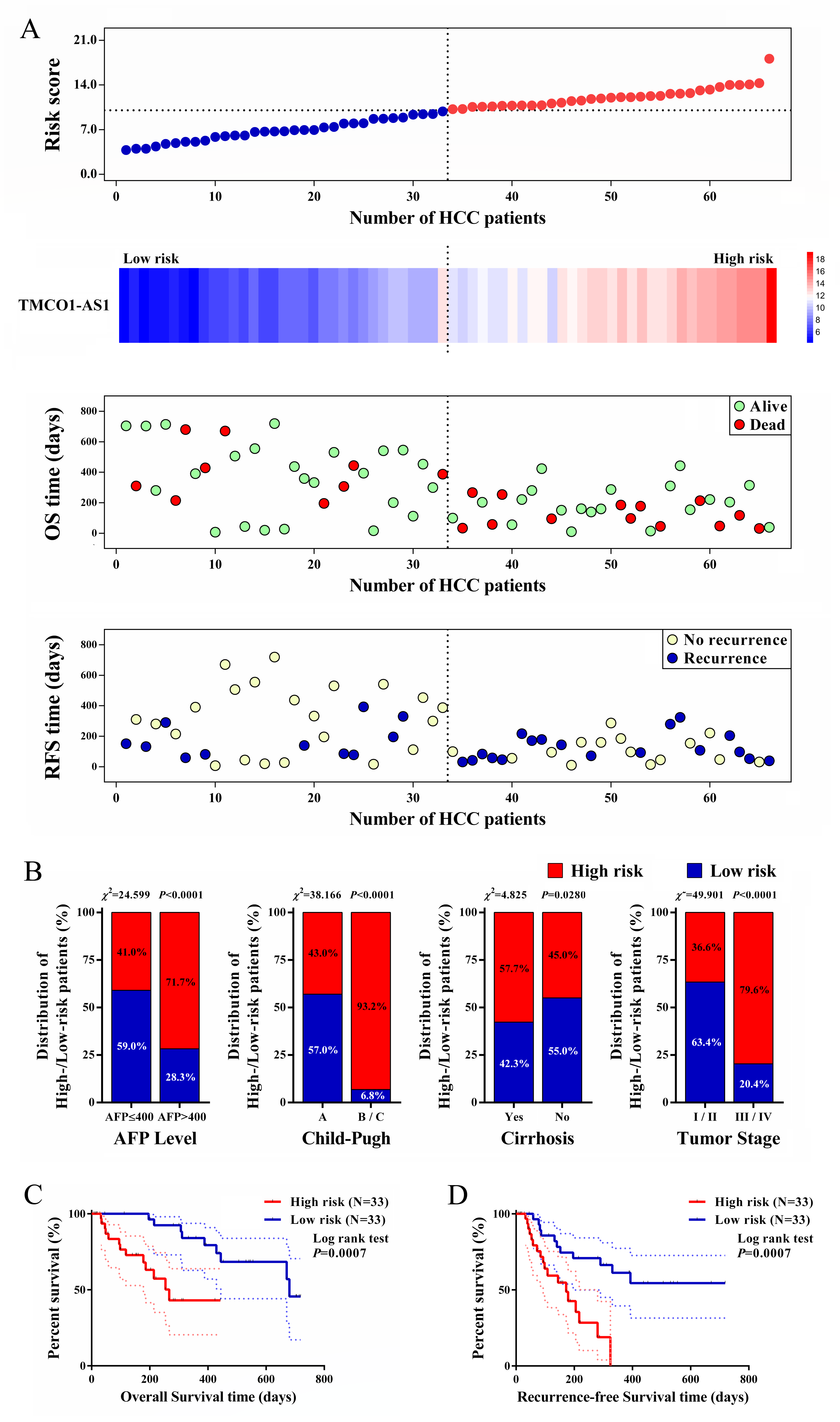

Supplement: Supplementary file 1 [file Image1.TIF]
