# Supplementary material for: High Expression of Long Non-Coding RNA TMCO1-AS1 is Associated With Poor Prognosis of Hepatocellular Carcinoma
Source: Front Mol Biosci. 2022 Jan 24;9:814058. doi: 10.3389/fmolb.2022.814058 (PMC8819098; doi:10.3389/fmolb.2022.814058)
Supplement: Supplementary file 2 [file DataSheet1.docx]

**Supplementary Materials**

**TABLE S1 |** Top 100 differentially expressed lncRNAs between HCC and adjacent tissues.

| **lncRNA** | **Ensembl ID** | **Location** | **Log_2_(FC)** | ***P* value** | **Regulation** |
| --- | --- | --- | --- | --- | --- |
| HAGLR | ENSG00000224189 | chr2:176173195-176188958 | 4.145624 | 3.6551×10^-21^ | Up |
| ST8SIA6-AS1 | ENSG00000204832 | chr10:17386936-17413503 | 3.709205 | 3.1034×10^-13^ | Up |
| HAND2-AS1 | ENSG00000237125 | chr4:173527270-173591324 | 3.124883 | 1.4252×10^-14^ | Down |
| AC092384.2 | ENSG00000259881 | chr16:88881038-88887136 | 2.843136 | 2.9149×10^-12^ | Down |
| FENDRR | ENSG00000268388 | chr16:86474529-86509099 | 2.803229 | 1.3238×10^-15^ | Down |
| TEX41 | ENSG00000226674 | chr2:144667967-145262988 | 2.767485 | 1.9158×10^-8^ | Up |
| AL365181.2 | ENSG00000272068 | chr1:156637783-156641004 | 2.746401 | 1.2196×10^-8^ | Up |
| AC004160.1 | ENSG00000230333 | chr7:11180902-11520175 | 2.683353 | 4.7639×10^-12^ | Down |
| C17orf82 | ENSG00000187013 | chr17:61411751-61413280 | 2.660255 | 2.1261×10^-17^ | Up |
| DUXAP8 | ENSG00000206195 | chr22:15784959-15829984 | 2.654778 | 3.2299×10^-10^ | Up |
| DDX11-AS1 | ENSG00000245614 | chr12:31020763-31073847 | 2.559893 | 3.7727×10^-19^ | Up |
| AC104809.2 | ENSG00000233392 | chr2:240954617-240967451 | 2.531887 | 8.2710×10^-14^ | Down |
| FAM99A | ENSG00000205866 | chr11:1665597-1667856 | 2.506867 | 2.9946×10^-10^ | Down |
| AF165147.1 | ENSG00000232855 | chr21:28439346-28674848 | 2.472803 | 3.2540×10^-13^ | Down |
| AL365181.3 | ENSG00000272405 | chr1:156641666-156644887 | 2.453332 | 5.5451×10^-9^ | Up |
| TMCO1-AS1 | ENSG00000224358 | chr1:165768929-165775176 | 2.440635 | 1.2378×10^-18^ | Up |
| AC147651.1 | ENSG00000223855 | chr7:520391-525232 | 2.440572 | 6.0700×10^-15^ | Up |
| LINC01093 | ENSG00000249173 | chr4:184893871-184899454 | 2.399192 | 1.7589×10^-14^ | Down |
| LY6E-DT | ENSG00000247317 | chr8:142981738-143018437 | 2.392682 | 1.5873×10^-9^ | Down |
| AC004540.2 | ENSG00000225792 | chr7:26372144-26376701 | 2.392653 | 8.7522×10^-12^ | Down |
| AC239809.3 | ENSG00000227733 | chr1:148159213-148254985 | 2.366889 | 1.3301×10^-8^ | Up |
| LINC00907 | ENSG00000267586 | chr18:42159283-42691422 | 2.366726 | 1.7376×10^-7^ | Down |
| ZNF252P-AS1 | ENSG00000255559 | chr8:145002811-145006046 | 2.320848 | 2.2835×10^-15^ | Up |
| AL109615.3 | ENSG00000237686 | chr6:43995723-44074652 | 2.314814 | 3.9044×10^-10^ | Up |
| U73166.1 | ENSG00000230454 | chr3:50260303-50263358 | 2.311936 | 1.3238×10^-15^ | Up |
| HHIP-AS1 | ENSG00000248890 | chr4:144642922-144661357 | 2.271218 | 3.2119×10^-13^ | Down |
| AL162431.1 | ENSG00000243155 | chr1:180944042-180976482 | 2.251487 | 1.6769×10^-16^ | Up |
| AL590666.2 | ENSG00000229953 | chr1:156646507-156661424 | 2.24849 | 6.1379×10^-10^ | Up |
| CRNDE | ENSG00000245694 | chr16:54918863-54929189 | 2.177753 | 2.9946×10^-10^ | Up |
| AC091057.1 | ENSG00000187951 | chr15:30624494-30772993 | 2.167247 | 2.7872×10^-13^ | Up |
| CAPN10-DT | ENSG00000260942 | chr2:240582700-240586699 | 2.157882 | 1.9958×10^-13^ | Up |
| AL353708.3 | ENSG00000272906 | chr1:179881607-179882595 | 2.156151 | 3.9864×10^-15^ | Up |
| LINC00511 | ENSG00000227036 | chr17:72323123-72640472 | 2.151482 | 4.2241×10^-7^ | Up |
| AL121906.2 | ENSG00000275223 | chr20:33655701-33656423 | 2.130851 | 1.9664×10^-14^ | Up |
| AL136162.1 | ENSG00000271888 | chr6:15243923-15245000 | 2.113696 | 9.1133×10^-17^ | Up |
| LINC00607 | ENSG00000235770 | chr2:215611563-215843722 | 2.107095 | 5.2304×10^-8^ | Up |
| TM4SF19-AS1 | ENSG00000235897 | chr3:196318330-196325570 | 2.073071 | 1.0882×10^-14^ | Up |
| LINC01694 | ENSG00000233922 | chr21:45593654-45603056 | 2.04546 | 6.2057×10^-8^ | Up |
| AC092119.2 | ENSG00000274460 | chr16:21950218-21951708 | 2.031064 | 6.6581×10^-13^ | Up |
| MAFG-DT | ENSG00000265688 | chr17:81927829-81930753 | 2.029636 | 1.1599×10^-14^ | Up |
| LINC01426 | ENSG00000234380 | chr21:34745757-34784886 | 2.029232 | 2.7246×10^-7^ | Up |
| HCG25 | ENSG00000232940 | chr6:33249534-33254989 | 2.026674 | 1.4030×10^-18^ | Up |
| AC087392.1 | ENSG00000262003 | chr17:909632-911212 | 2.025596 | 3.9566×10^-9^ | Down |
| AC092171.2 | ENSG00000230733 | chr7:5475804-5479811 | 2.019572 | 3.6551×10^-21^ | Up |
| DNM1P35 | ENSG00000246877 | chr15:75727670-75738623 | 2.004716 | 6.7583×10^-12^ | Up |
| RP11-98G7.1 | ENSG00000236947 | chr1:156446412-156456577 | 2.002646 | 1.0491×10^-10^ | Up |
| AC102953.2 | ENSG00000273230 | chr7:1464497-1467522 | 1.998942 | 1.4505×10^-13^ | Up |
| MYLK-AS1 | ENSG00000239523 | chr3:123585542-123644568 | 1.980197 | 3.9984×10^-20^ | Up |
| AC108751.4 | ENSG00000243885 | chr3:149384179-149385800 | 1.957285 | 3.9194×10^-12^ | Up |
| AC010719.1 | ENSG00000270933 | chr7:25948657-25949403 | 1.954529 | 4.8316×10^-13^ | Up |
| FAM99B | ENSG00000205865 | chr11:1683269-1685629 | 1.951257 | 1.7104×10^-9^ | Down |
| PURPL | ENSG00000250337 | chr5:27472292-27496401 | 1.948863 | 1.0134×10^-6^ | Up |
| LINC00853 | ENSG00000224805 | chr1:47179250-47180339 | 1.94359 | 3.2335×10^-15^ | Up |
| LINC01311 | ENSG00000260924 | chr22:19171395-19172839 | 1.93082 | 7.1665×10^-16^ | Up |
| DIO3OS | ENSG00000258498 | chr14:101552221-101560431 | 1.929345 | 2.1707×10^-6^ | Down |
| AC079061.1 | ENSG00000248050 | chr8:109644115-109648084 | 1.928367 | 3.9757×10^-9^ | Down |
| AC132192.2 | ENSG00000268403 | chr11:9459556-9460702 | 1.92764 | 7.1665×10^-16^ | Up |
| SNHG4 | ENSG00000281398 | chr5:139274102-139283244 | 1.927316 | 1.5927×10^-11^ | Up |
| LINC02600 | ENSG00000250986 | chr4:3758748-3763390 | 1.89846 | 4.7380×10^-7^ | Up |
| AL121832.3 | ENSG00000275437 | chr20:62402236-62405935 | 1.88489 | 1.4240×10^-10^ | Up |
| FAM182B | ENSG00000175170 | chr20:25763466-25868225 | 1.875717 | 4.4278×10^-9^ | Up |
| AC111000.4 | ENSG00000250696 | chr4:69182100-69216766 | 1.871241 | 2.7274×10^-10^ | Up |
| AC012313.8 | ENSG00000269473 | chr19:58440448-58445849 | 1.869944 | 6.8480×10^-11^ | Up |
| BAALC-AS1 | ENSG00000247081 | chr8:103156990-103298772 | 1.868596 | 6.9859×10^-11^ | Up |
| ZKSCAN2-DT | ENSG00000274925 | chr16:25257952-25261066 | 1.866456 | 1.1060×10^-13^ | Up |
| AC010280.2 | ENSG00000248884 | chr5:68430427-68434481 | 1.855485 | 2.6436×10^-12^ | Down |
| LNCOC1 | ENSG00000253741 | chr8:142702252-142726973 | 1.849944 | 3.2197×10^-9^ | Up |
| TMCC1-AS1 | ENSG00000271270 | chr3:129893871-129918575 | 1.840413 | 4.7950×10^-16^ | Up |
| FZD10-AS1 | ENSG00000250208 | chr12:130144315-130162256 | 1.835742 | 1.4543×10^-6^ | Up |
| TDRKH-AS1 | ENSG00000203288 | chr1:151790804-151794402 | 1.823979 | 2.2322×10^-11^ | Up |
| AC107959.3 | ENSG00000253616 | chr8:23071377-23074488 | 1.821432 | 5.7856×10^-8^ | Up |
| AC245060.6 | ENSG00000279278 | chr22:22264601-22273020 | 1.821009 | 3.5298×10^-9^ | Up |
| AL391056.1 | ENSG00000227619 | chr9:129575409-129584556 | 1.813388 | 2.8741×10^-5^ | Up |
| AC006538.1 | ENSG00000261342 | chr19:2727743-2729327 | 1.813218 | 8.1123×10^-11^ | Up |
| RP11-529E10.6 | ENSG00000261643 | chr4:3503597-3504457 | 1.811437 | 1.0362×10^-11^ | Up |
| AL121772.1 | ENSG00000274414 | chr20:25239007-25245229 | 1.805849 | 2.8915×10^-9^ | Up |
| DLG5-AS1 | ENSG00000233871 | chr10:77927372-77929824 | 1.802698 | 3.5610×10^-15^ | Up |
| LINC00702 | ENSG00000233117 | chr10:4201141-4243912 | 1.797998 | 2.7691×10^-13^ | Up |
| AC010201.2 | ENSG00000271327 | chr12:89367807-89369301 | 1.795147 | 2.4008×10^-12^ | Up |
| AC237221.1 | ENSG00000260653 | chr7:57404771-57416203 | 1.794783 | 7.8792×10^-5^ | Up |
| AC016596.1 | ENSG00000227908 | chr5:55995167-56003649 | 1.794126 | 3.0442×10^-10^ | Up |
| AL021707.2 | ENSG00000228274 | chr22:38667585-38681820 | 1.787039 | 1.2179×10^-13^ | Up |
| AC139100.1 | ENSG00000267251 | chr18:80183680-80202992 | 1.784584 | 1.5673×10^-11^ | Up |
| AL158212.3 | ENSG00000260917 | chr10:112823490-112827726 | 1.781847 | 1.2191×10^-12^ | Up |
| ZFPM2-AS1 | ENSG00000251003 | chr8:105780246-106060524 | 1.780709 | 1.3760×10^-5^ | Up |
| GAPLINC | ENSG00000266835 | chr18:3466250-3478978 | 1.780575 | 2.1212×10^-9^ | Up |
| AC036108.2 | ENSG00000261054 | chr15:99128832-99131806 | 1.780554 | 1.1548×10^-8^ | Up |
| LINC00665 | ENSG00000232677 | chr19:36313067-36331718 | 1.760218 | 2.1562×10^-9^ | Up |
| LINC01703 | ENSG00000225518 | chr1:226083639-226090292 | 1.756194 | 6.0700×10^-15^ | Up |
| AC013731.1 | ENSG00000270557 | chr2:157877683-157878565 | 1.756104 | 1.2715×10^-11^ | Up |
| AL139385.1 | ENSG00000275880 | chr13:110613082-110616353 | 1.75017 | 6.2801×10^-9^ | Down |
| AC013275.1 | ENSG00000231013 | chr2:119476448-119487346 | 1.743286 | 3.1948×10^-8^ | Up |
| AL357079.1 | ENSG00000237950 | chr1:43944370-43946551 | 1.7409 | 2.9896×10^-12^ | Up |
| LINC02709 | ENSG00000245522 | chr11:9754770-9759533 | 1.73741 | 1.2614×10^-10^ | Up |
| AC105219.1 | ENSG00000181097 | chr8:143696154-143698413 | 1.737368 | 4.0100×10^-13^ | Up |
| LINC00950 | ENSG00000281205 | chr9:35858738-35865518 | 1.73493 | 9.6315×10^-12^ | Up |
| AC099850.4 | ENSG00000265415 | chr17:59202677-59203829 | 1.724867 | 1.3787×10^-12^ | Up |
| AC020907.1 | ENSG00000179066 | chr19:35105969-35107318 | 1.722553 | 3.7342×10^-10^ | Up |
| AC138356.1 | ENSG00000183154 | chr8:37734761-37737426 | 1.721122 | 6.4853×10^-12^ | Down |
| C1QTNF1-AS1 | ENSG00000265096 | chr17:79019209-79027655 | 1.717592 | 2.4605×10^-6^ | Up |

**TABLE S2 |** Univariate regression analysis of the top 100 differential expressed lncRNAs.

| **lncRNA** | **OS** | |  | **RFS** | |
| --- | --- | --- | --- | --- | --- |
|  | HR | *P* value |  | HR | *P* value |
| HAGLR | 1.0487 | 0.4650 |  | 1.0484 | 0.3905 |
| ST8SIA6-AS1 | 1.0818 | 0.2019 |  | 1.0694 | 0.2040 |
| HAND2-AS1 | 0.9164 | 0.7345 |  | 0.6967 | 0.1764 |
| AC092384.2 | 0.7184 | 0.0870 |  | 0.9681 | 0.8140 |
| FENDRR | 0.5867 | 0.4699 |  | 1.0800 | 0.9064 |
| TEX41 | 1.3192 | 0.1233 |  | 1.0515 | 0.7679 |
| AL365181.2 | 1.0011 | 0.9901 |  | 1.0728 | 0.3639 |
| AC004160.1 | 0.6772 | 0.1408 |  | 0.6429 | 0.0534 |
| C17orf82 | 1.0219 | 0.8895 |  | 1.2078 | 0.1326 |
| DUXAP8 | 1.6736 | 0.0002 |  | 1.3553 | 0.0182 |
| DDX11-AS1 | 2.2437 | 0.0006 |  | 2.0290 | 0.0005 |
| AC104809.2 | 0.9292 | 0.5299 |  | 1.1591 | 0.0580 |
| FAM99A | 0.8873 | 0.0172 |  | 0.9635 | 0.3520 |
| AF165147.1 | 0.7226 | 0.7034 |  | 0.7380 | 0.6808 |
| AL365181.3 | 1.0012 | 0.9834 |  | 0.9876 | 0.8013 |
| TMCO1-AS1 | 1.9315 | 0.0087 |  | 1.9614 | 0.0026 |
| AC147651.1 | 1.0853 | 0.5496 |  | 1.0908 | 0.4416 |
| LINC01093 | 0.9008 | 0.0797 |  | 0.9356 | 0.1613 |
| LY6E-DT | 0.9344 | 0.7106 |  | 0.9961 | 0.9805 |
| AC004540.2 | 1.0100 | 0.9191 |  | 1.0066 | 0.9392 |
| AC239809.3 | 1.1803 | 0.3149 |  | 0.9491 | 0.7768 |
| LINC00907 | 0.4735 | 0.3748 |  | 0.5361 | 0.3335 |
| ZNF252P-AS1 | 2.6251 | 0.0154 |  | 2.1396 | 0.0266 |
| AL109615.3 | 1.1662 | 0.0921 |  | 1.1413 | 0.0875 |
| U73166.1 | 1.1774 | 0.4724 |  | 1.7979 | 0.0012 |
| HHIP-AS1 | 1.0453 | 0.7643 |  | 0.9027 | 0.5047 |
| AL162431.1 | 1.4616 | 0.0395 |  | 1.3526 | 0.0503 |
| AL590666.2 | 1.0272 | 0.7015 |  | 0.9798 | 0.7266 |
| CRNDE | 1.1332 | 0.1360 |  | 1.1659 | 0.0326 |
| AC091057.1 | 1.6033 | 0.0138 |  | 1.8155 | 0.0002 |
| CAPN10-DT | 1.4231 | 0.0890 |  | 1.5870 | 0.0035 |
| AL353708.3 | 1.3671 | 0.0819 |  | 1.4585 | 0.0181 |
| LINC00511 | 1.2673 | 0.0155 |  | 1.1784 | 0.0757 |
| AL121906.2 | 1.1270 | 0.3459 |  | 1.2873 | 0.0166 |
| AL136162.1 | 1.4546 | 0.0526 |  | 1.7153 | 0.0007 |
| LINC00607 | 1.3199 | 0.0304 |  | 1.0730 | 0.5657 |
| TM4SF19-AS1 | 1.7345 | 0.0151 |  | 1.8670 | 0.0008 |
| LINC01694 | 1.3436 | 0.0675 |  | 1.2843 | 0.0911 |
| AC092119.2 | 1.1031 | 0.6333 |  | 1.4626 | 0.0148 |
| MAFG-DT | 1.2993 | 0.0007 |  | 1.1145 | 0.1335 |
| LINC01426 | 0.9772 | 0.7955 |  | 1.1253 | 0.1153 |
| HCG25 | 1.5904 | 0.0213 |  | 1.5162 | 0.0198 |
| AC087392.1 | 0.8687 | 0.1802 |  | 0.8988 | 0.1955 |
| AC092171.2 | 1.2608 | 0.0199 |  | 1.1625 | 0.0711 |
| DNM1P35 | 1.0435 | 0.8411 |  | 0.9572 | 0.8119 |
| RP11-98G7.1 | 1.3492 | 0.0088 |  | 1.2452 | 0.0291 |
| AC102953.2 | 1.3190 | 0.0784 |  | 1.4270 | 0.0057 |
| MYLK-AS1 | 1.4180 | 0.0072 |  | 1.1990 | 0.1286 |
| AC108751.4 | 1.1793 | 0.2532 |  | 1.1508 | 0.2682 |
| AC010719.1 | 1.1119 | 0.2785 |  | 1.1841 | 0.0414 |
| FAM99B | 0.8566 | 0.0399 |  | 0.9593 | 0.4874 |
| PURPL | 0.9698 | 0.7635 |  | 1.1202 | 0.2007 |
| LINC00853 | 1.1492 | 0.1161 |  | 1.0099 | 0.9007 |
| LINC01311 | 1.2688 | 0.1653 |  | 1.2304 | 0.1634 |
| DIO3OS | 0.7653 | 0.0319 |  | 0.8759 | 0.1715 |
| AC079061.1 | 0.7770 | 0.1184 |  | 1.0685 | 0.5492 |
| AC132192.2 | 1.2002 | 0.1745 |  | 1.3375 | 0.0095 |
| SNHG4 | 1.6400 | 0.0001 |  | 1.3592 | 0.0086 |
| LINC02600 | 1.2954 | 0.1152 |  | 1.2364 | 0.1383 |
| AL121832.3 | 1.8330 | 0.0625 |  | 2.5980 | 0.0001 |
| FAM182B | 2.0725 | 0.0007 |  | 1.1443 | 0.6199 |
| AC111000.4 | 1.1506 | 0.1625 |  | 1.1573 | 0.0683 |
| AC012313.8 | 7.3168 | 0.0003 |  | 4.2949 | 0.0037 |
| BAALC-AS1 | 1.3425 | 0.2341 |  | 1.1417 | 0.5537 |
| ZKSCAN2-DT | 1.1699 | 0.4610 |  | 1.6549 | 0.0027 |
| AC010280.2 | 0.7622 | 0.0119 |  | 0.9287 | 0.3585 |
| LNCOC1 | 0.7580 | 0.5442 |  | 1.1019 | 0.7759 |
| TMCC1-AS1 | 2.4929 | 0.0000 |  | 1.6616 | 0.0019 |
| FZD10-AS1 | 1.3852 | 0.4913 |  | 1.3379 | 0.5500 |
| TDRKH-AS1 | 1.5146 | 0.0092 |  | 1.2630 | 0.1065 |
| AC107959.3 | 1.4977 | 0.0001 |  | 1.1881 | 0.0704 |
| AC245060.6 | 1.9052 | 0.1208 |  | 2.3240 | 0.0122 |
| AL391056.1 | 1.0809 | 0.3323 |  | 1.0500 | 0.5022 |
| AC006538.1 | 1.3516 | 0.0524 |  | 1.2525 | 0.1122 |
| RP11-529E10.6 | 1.1803 | 0.1898 |  | 1.1072 | 0.3458 |
| AL121772.1 | 1.1866 | 0.5128 |  | 1.6045 | 0.0337 |
| DLG5-AS1 | 1.1698 | 0.1110 |  | 1.0229 | 0.7943 |
| LINC00702 | 0.4250 | 0.0615 |  | 0.8289 | 0.5976 |
| AC010201.2 | 1.2930 | 0.1957 |  | 1.7904 | 0.0003 |
| AC237221.1 | 1.3498 | 0.0077 |  | 1.0649 | 0.5618 |
| AC016596.1 | 1.1633 | 0.4472 |  | 1.3582 | 0.0419 |
| AL021707.2 | 1.3713 | 0.1711 |  | 1.4964 | 0.0386 |
| AC139100.1 | 0.9273 | 0.6844 |  | 1.0154 | 0.9154 |
| AL158212.3 | 1.3870 | 0.0797 |  | 1.2707 | 0.1109 |
| ZFPM2-AS1 | 1.2887 | 0.0001 |  | 1.1569 | 0.0117 |
| GAPLINC | 0.9417 | 0.5254 |  | 0.9667 | 0.6827 |
| AC036108.2 | 1.1255 | 0.4681 |  | 1.4156 | 0.0038 |
| LINC00665 | 1.1949 | 0.0227 |  | 1.0741 | 0.2945 |
| LINC01703 | 1.2099 | 0.0527 |  | 1.1522 | 0.0993 |
| AC013731.1 | 1.3137 | 0.1497 |  | 1.5029 | 0.0094 |
| AL139385.1 | 0.8314 | 0.2826 |  | 0.9280 | 0.5753 |
| AC013275.1 | 1.1388 | 0.0702 |  | 1.1281 | 0.0543 |
| AL357079.1 | 1.6203 | 0.0046 |  | 1.4586 | 0.0111 |
| LINC02709 | 1.8268 | 0.0004 |  | 1.5052 | 0.0145 |
| AC105219.1 | 1.1414 | 0.6863 |  | 1.4960 | 0.1177 |
| LINC00950 | 1.0105 | 0.9777 |  | 1.4050 | 0.3030 |
| AC099850.4 | 1.3231 | 0.0003 |  | 1.2307 | 0.0015 |
| AC020907.1 | 1.0570 | 0.5809 |  | 1.1783 | 0.0471 |
| AC138356.1 | 0.6444 | 0.0404 |  | 0.7601 | 0.1095 |
| C1QTNF1-AS1 | 1.0034 | 0.9703 |  | 0.8382 | 0.0493 |

**TABLE S3 |** Univariate regression analysis of clinicopathological characteristics for OS and DFS in patients from TCGA.

| Clinicopathological Characteristics | | OS | | |  | RFS | | |
| --- | --- | --- | --- | --- | --- | --- | --- | --- |
|  |  | Coef | HR (95% CI) | *P* value^a^ |  | Coef | HR (95% CI) | *P* value^a^ |
| Age(years) | >60 VS. ≤60 | 0.2964 | 1.3450 (0.8996–2.0109) | 0.1486 |  | -0.0222 | 0.9781 (0.7015-1.3638) | 0.8960 |
| Gender | Male VS. Female | -0.2036 | 0.8158 (0.5464-1.2181) | 0.3195 |  | -0.1220 | 0.8852 (0.6272-1.2493) | 0.4878 |
| AFP(µg/L) | >400 VS. ≤400 | 0.3687 | 1.4458 (0.9606-2.1762) | 0.0772 |  | 0.1123 | 1.1189 (0.7746-1.6161) | 0.5494 |
| Hepatitis virus infection | Yes VS. No | -0.4327 | 0.6488 (0.4339-0.9701) | 0.0351* |  | -0.0357 | 0.9649 (0.6923-1.3449) | 0.8331 |
| Alcoholic hepatitis | Yes VS. No | -0.0354 | 0.9652 (0.6318-1.4745) | 0.8699 |  | 0.1056 | 0.8998 (0.6281-1.2891) | 0.5649 |
| Child-Pugh | B/C VS. A | 0.6072 | 1.8353 (1.1234-2.9983) | 0.0153* |  | 0.3386 | 1.4029 (0.8717-2.2581) | 0.1632 |
| Cirrhosis | Yes VS. No | 0.1821 | 1.1997 (0.8036-1.7912) | 0.3732 |  | 0.1345 | 1.1440 (0.8169-1.6020) | 0.4336 |
| Vascular invasion | Yes VS. No | 0.3190 | 1.3758 (0.9177-2.0625) | 0.1225 |  | 0.5535 | 1.7392 (1.2420-2.4356) | 0.0013* |
| Tumor stage | III/IV VS. I/II | 0.8130 | 2.2547 (1.5036-3.3809) | <0.0001* |  | 0.8497 | 2.3390 (1.6334-3.3494) | <0.0001* |
| Tumor differentiation | G3/G4 VS. G1/G2 | 0.1661 | 1.1807 (0.7939-1.7559) | 0.4121 |  | 0.1933 | 1.2132 (0.8671-1.6975) | 0.2593 |
| TMCO1-AS1 expression | High VS. Low | 0.5378 | 1.7123 (1.1513-2.5464) | 0.0079* |  | 0.4363 | 1.5469 (1.1083-2.1591) | 0.0103* |

^a^Univariate regression, **P* < 0.05.

**TABLE S4 |** Multivariate regression analysis of clinicopathological characteristics for OS and DFS in patients from TCGA.

| Clinicopathological Characteristics | | OS | | |  | RFS | | |
| --- | --- | --- | --- | --- | --- | --- | --- | --- |
|  |  | Coef | HR (95% CI) | *P* value^a^ |  | Coef | HR (95% CI) | *P* value^a^ |
| Child-Pugh | B/C VS. A | 0.5241 | 1.6889 (1.0297-2.7702) | 0.0379* |  | / | / | / |
| Vascular invasion | Yes VS. No | / | / | / |  | 0.3666 | 1.4428 (1.0195-2.0419) | 0.0385* |
| Tumor stage | III/IV VS. I/II | 0.7368 | 2.0893 (1.3858-3.1501) | 0.0004* |  | 0.7337 | 2.0828 (1.4410-3.0103) | <0.0001* |
| TMCO1-AS1 expression | High VS. Low | 0.4657 | 1.5931 (1.0672-2.3780) | 0.0227* |  | 0.3448 | 1.4117 (1.0084-1.9764) | 0.0446* |

^a^Multivariate regression, **P* < 0.05.
